# Green Synthesis of NiFe_2_O_4_ Nano-Spinel Oxide-Decorated Carbon Nanotubes for Efficient Capacitive Performance—Effect of Electrolyte Concentration

**DOI:** 10.3390/nano13192643

**Published:** 2023-09-26

**Authors:** Ali H. Bashal, Mahmoud A. Hefnawy, Hoda A. Ahmed, Mohamed A. El-Atawy, Rami Adel Pashameah, Shymaa S. Medany

**Affiliations:** 1 Chemistry Department, Faculty of Science at Yanbu, Taibah University, Yanbu 46423, Saudi Arabia; 2 Chemistry Department, Faculty of Science, Cairo University, Giza 12613, Egypt; 3 Chemistry Department, Faculty of Science, Alexandria University, Ibrahemia, Alexandria 21321, Egypt; 4 Department of Chemistry, Faculty of Applied Science, Umm Al-Qura University, Makkah 24230, Saudi Arabia

**Keywords:** nickel–iron oxide, spinel oxide, capacitance, galvanostatic charging/discharging, electrochemical capacitor

## Abstract

Energy storage applications received great attention due to environmental aspects. A green method was used to prepare a composite of nickel–iron-based spinel oxide nanoparticle@CNT. The prepared materials were characterized by different analytical methods like X-ray diffraction, X-ray photon spectroscopy (XPS), scanning electron microscopy (SEM), and transmitted electron microscopy (TEM). The synergistic effect between nickel–iron oxide and carbon nanotubes was characterized using different electrochemical methods like cyclic voltammetry (CV), galvanostatic charging/discharging (GCD), and electrochemical impedance spectroscopy (EIS). The capacitances of the pristine NiFe_2_O_4_ and NiFe_2_O_4_@CNT were studied in different electrolyte concentrations. The effect of ${OH}^{-}$ concentrations was studied for modified and non-modified surfaces. Furthermore, the specific capacitance was estimated for pristine and modified NiFe_2_O_4_ at a wide current range (5 to 17 A g^−1^). Thus, the durability of different surfaces after 2000 cycles was studied, and the capacitance retention was estimated as 78.8 and 90.1% for pristine and modified NiFe_2_O_4_. On the other hand, the capacitance rate capability was observed as 65.1% (5 to 17 A g^−1^) and 62.4% (5 to 17 A g^−1^) for NiFe_2_O_4_ and NiFe_2_O_4_@CNT electrodes.

## 1. Introduction

Renewable energy is becoming an increasingly important part of our lives as we strive to reduce our dependence on fossil fuels. With the help of renewable energy sources, such as solar, wind, and geothermal power, we can reduce our carbon footprint and create a more sustainable future. Renewable energy also has the potential to create jobs and stimulate economic growth in communities that are heavily reliant on fossil fuels. As we move toward a greener future, it is important to understand the various used cases of renewable energy and how they can benefit individuals and society [1,2,3,4].

A capacitor is an electrical component that stores energy as an electric field. It is used in many electronic circuits to store and release energy when needed [5,6,7,8,9]. Capacitors are widely used in various applications, from power supplies to audio equipment. They also filter out noise and interference from signals and provide a steady voltage supply for sensitive components. The capacity of a capacitor is determined by its size and fabricated material, so choosing the right type for your application is essential [10,11,12,13,14].

The nickel-based modified surface has recently been reported as an efficient material for electrochemical applications in basic media because it generates NiOOH electroactive species [15,16]. Thus, Ni-based materials are reported for applications like fuel cells, electrochemical sensors, and batteries [17,18,19,20,21,22,23,24,25,26].

Spinel oxides with a typical structure of AB_2_O_4_ (where A and B are transition metals) are durable and robust materials for chemical and thermal changes that are widely used in different applications [27,28]. Metal spinel oxides may be classified into three categories: monometallic spinel oxides, bimetallic spinel oxides, and polymetallic spinel oxides. Magnetic spinel oxides have favorable characteristics in terms of their magnetic, electrical, and catalytic attributes, rendering them extensively employed in many domains such as magnetic materials, electronic devices, and catalysis, among others [29].

Spinel ferrites have garnered a lot of interest recently because of their many redox states, excellent electrochemical stability, and pseudo capacitive behavior. The spinel ferrites outperform other metal oxides in terms of operation voltage and theoretical capacity. However, because of its high surface energy and significant particle aggregation, its practical capacitance and cycling characteristic still require improvement, and creating pertinent composites appears to be a potential path to improving the electrochemical performance.

In recent years, NiFe_2_O_4_ has been employed for various applications like electrochemical sensors [23,30,31], water splitting [32,33,34], solar cells [35], and supercapacitors [36,37,38].

Carbon support materials were reported extensively for catalysis applications. High surface area, suitable electrical conductivity, and low toxicity are considered the most important points for using carbon materials to support modified surfaces. Different carbon materials were reported as proper support for transition metals-based modified surfaces like carbon nanotubes (CNTs) [39,40], graphene [41,42,43], graphene oxides [44], conducting polymer [45], chitosan [46,47], and porous carbon [48]. With their superior electronic conductivity and robust mechanical qualities, carbon nanotubes are frequently used as electrode materials for energy storage systems [49,50,51].

Consequently, several NiFe_2_O_4_-based surfaces are used for high capacitance application such as NiFe_2_O_4_/MoS_2_, rGO-NiFe_2_O_4_, and PANI-NiFe_2_O_4_ [52,53,54].

However, different approaches could be used for preparation of spinel oxides like hydrothermal, sol-gel, and coprecipitation. Thus, green approaches were recently reported in the literature to increase the sustainability of the preparation steps.

Several green plant extracts, including Terminalia Catappa, Tamarindus Indica seeds, and Urtica, have been widely reported for the production of nickel ferrite [55,56,57]. The botanical extract comprises various organic compounds that act as green-reducing and capping agents, such as phenolic compounds, flavonoids, carotenoids, and vitamins. Green tea extract is a commonly utilized substance in the production of metals due to its inclusion of diverse reducing and capping agents such as enzymes, polyphenols, and amino acids.

The concentration of, herein, NiFe_2_O_4_-decorated carbon nanotube composites was examined for enhancing the capacitance, in addition to the role and advantages that carbon nanotubes can provide to capacitor designs. The nickel ferrite was prepared using green methods. A comparative study was performed between pristine NiFe_2_O_4_ and NiFe_2_O_4_@CNT. Different analytical approaches characterized the prepared materials. The effect of electrolyte concentration was studied, whereas galvanostatic charging/discharging was utilized at wide current density ranges. The durability of the electrode was investigated using repeated charging and discharging cycles. EIS was employed to determine the charge transfer resistance for pristine and modified NiFe_2_O_4_ surfaces for different electrolyte concentrations.

## 2. Experimental Section

### 2.1. Green Synthesis of NiFe_2_O_4_

The preparation of the green tea extract involved the boiling of green tea leaves in 100 mL of distilled water. Subsequently, the solution was subjected to cooling and filtration procedures, followed by transferal into a sterile container. The precursor salts of Fe(NO_3_)_3.9_H_2_O and Ni(NO_3_)_2.6_H_2_O were dissolved in a 2:1 molar ratio in 50 mL of deionized water. The process involved gradually adding green tea leaf extract into the precursor solutions, followed by heating to 80 °C with intense agitation. Subsequently, the fluid was subjected to thermal energy until a solid-like substance was produced. The gel underwent a drying process for a duration of two hours at a temperature of 150 °C in an oven. The specimens underwent a four-hour annealing process at a temperature of 600 °C.

### 2.2. Electrode Preparation and Electrochemical Measurements

The experimental setup utilized a working electrode in the form of a glassy carbon electrode (GC) with a diameter of 3 mm and a surface area of 0.0707 cm^2^. The initial step involved the use of a mild emery paper for polishing, followed by a thorough cleansing process utilizing ethanol and double-distilled water. Two catalyst inks were produced through the dispersion of 60 mg of pristine NiFe_2_O_4_ and 60 mg of NiFe_2_O_4_@CNT (with a NiFe_2_O_4_: CNT ratio of 1:1) in a solution comprising 1 mL of ethanol and 1 mL of 5 wt% Nafion. This facilitated the uniform distribution of NiFe_2_O_4_ on every electrode. Subsequently, a volume of 50 µL of ink was deposited onto the surface of the glassy carbon electrode and subjected to desiccation overnight. The catalyst loading was determined to be 1.5 mg. Consequently, the modified surfaces that were prepared, namely pristine NiFe_2_O_4_ and NiFe_2_O_4_@CNT, were denoted as NFO and NFO@CNT, respectively.

Autolab PGSTAT128N was utilized to acquire data through cyclic voltammetry, electrochemical impedance spectroscopy, and galvanostatic charging/discharging. The electrochemistry tests and impedance spectrum fitting were conducted using NOVA software (Version 2.1, Metrohm Autolab, Utrecht, The Netherlands). A three-electrode cell was employed, with Ag/AgCl/KCl (sat.) serving as the reference electrode, Pt wire as the counter electrode, and GC/NiFe_2_O_4_ and GC/NiFe_2_O_4_@CNT as the working electrodes. The computation of all conceivable values in this study was executed utilizing reference electrodes of Ag/AgCl/KCl (sat.). The electrochemical impedance spectroscopy measurements were adjusted to a consistent AC voltage value by utilizing an AC voltage amplitude of 10 mV and a frequency range spanning from 10^4^ Hz to 0.1 Hz.

The following equation was used to compute the specific capacitances of supercapacitors during discharge:

(1) $$C=\frac{I\;∆T}{m\;∆V\;}$$

The specific capacitance (C) was determined using the discharging current (*I*), the time required for discharging (∆*T*), the mass of modified surface (*m*), and the potential window (∆*V*).

## 3. Results and Discussion

### 3.1. Material Characterization

**Figure 1:**
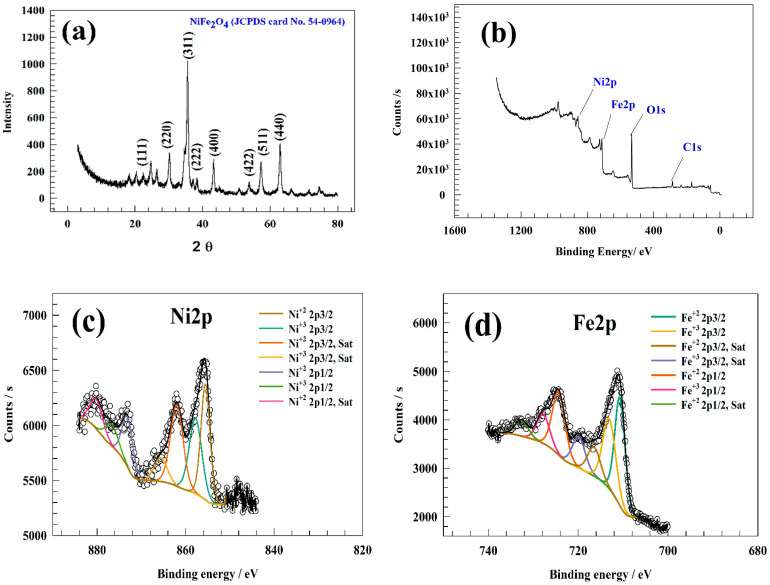

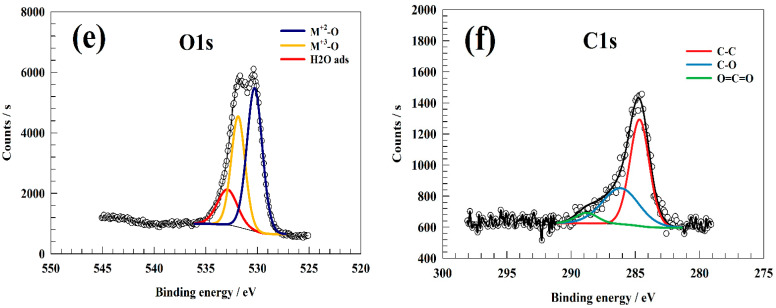
(**a**) XRD of NiFe_2_O_4_; XPS of NiFe_2_O_4_ (**b**) survey spectrum, spectra of (**c**) Ni2p, (**d**) Fe2p, (**e**) O1s, and (**f**) C1s. Black circle is the raw data (without modification), black frame is total peak without deconvolution.

The Powder X-ray diffraction technique characterized the chemical structure of the prepared NiFe_2_O_4_. An XRD chart of the NiFe_2_O_4_ is illustrated in Figure 1a. According to reference card JCPDS No.54-0964, seven characteristic peaks were observed for NiFe_2_O_4_ at 2*θ* = 22.5, 30.2, 35.3, 36.4, 43.2, 53.7, 57.4, and 63.2°, corresponding to miller indices (111), (220), (311), (222), (400), (422), (511), and (440), respectively. The estimated crystal system of NiFe_2_O_4_ is cubic with crystal point group m$\overset{-}{3}$m.

Furthermore, X-ray photon spectroscopy (XPS) characterized the oxidation states and types of bonds between atoms. Figure 1b shows an introductory survey of the NiFe_2_O_4_, which displays the presence of Ni, Fe, O, and C at a binding energy of 857.38, 712.35, 285.81, and 532.07 eV, respectively. The XPS spectra of the NiFe_2_O_4_ elements are displayed in Figure 1c–f. As shown in Figure 1c, Ni2P spectra were observed to have characteristic peaks at 855.5 and 857.76 eV, corresponding to 2p3/2 Ni^+2^ and Ni^+3^ peaks. The peaks observed at 862.1 and 865.62 were due to satellite of Ni 2p3/2 [58]. The peaks observed at 873.15, 876.67, and 880.14 eV were also attributed to Ni 2p1/2 and its satellites. Figure 1d displays the XPS spectra of the 2p core level of Fe. The spectra showed Fe2p signals at 710.69 and 713.08 eV, which are attributed to Fe2p3/2. Furthermore, peaks were observed at 716.47 and 719.88 eV for 2p3/2 satellites [59]. The peaks at binding energies of 724.34, 727.79, and 732.61 eV are attributed to Fe2p1/2 and its satellite.

The origin of oxygenated Ni and Fe bonds can be ascribed by the peaks at 530.28 and 531.87 eV in Figure 1e. The peak observed at a binding energy of 532.91 eV is attributed to the water molecules that were adsorbed on the surface of the catalyst [60]. Figure 1f depicts the C1s spectra. Three distinct peaks were detected in the C1s spectra at binding energies of 284.83, 285.32, and 288.58 electron volts. The spectral peaks detected at 284.83 and 285.32 electron volts are indicative of a carbonaceous layer that is typically present on the surfaces of air-exposed samples. The third peak observed at a binding energy of 288.58 eV indicates the existence of metal carbonate, as reported in previous studies [61,62].

**Figure 2:**
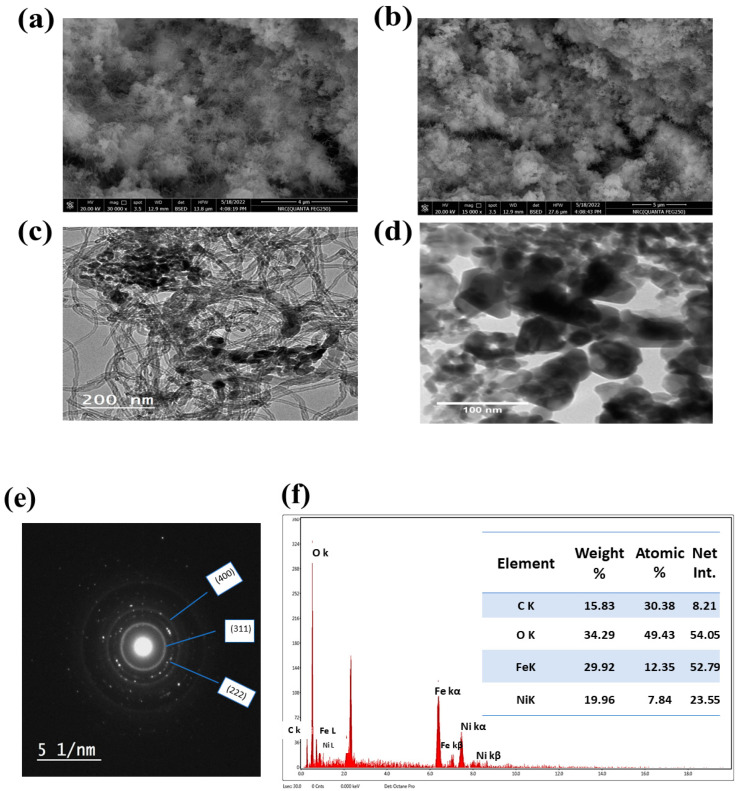
SEM of NiFe_2_O_4_@CNT with (**a**) before and (**b**) after stability test. (**c**) TEM of NiFe_2_O_4_@CNT, (**d**) TEM of pristine NiFe_2_O_4_, (**e**) diffraction pattern of NiFe_2_O_4_@CNT sample, and (**f**) EDAX and elemental analysis of NiFe_2_O_4_@CNT sample.

A scanning electron microscope characterized the surface morphology of the modified GC/NFO@CNT electrode. Figure 2a shows a modified GC/NFO@CNT. Thus, NiFe_2_O_4_ was observed to decorate the carbon nanotube. The cavity within the surface of NFO@CNT promotes the diffusion of O$H^{-}.$ The well distribution of NFO on the CNT surface led to higher efficiency. Additionally, the structure stability after cycling was characterized using SEM (see Figure 2b). The particle coagulated after cycling due to the deterioration of nickel ferrite after several Ni(OH)_2_/NiOOH conversions.

The morphological structure and nanoparticle distribution were characterized using a transmitted electron microscope (TEM). The TEM image of the NFO@CNT sample is represented in Figure 2c. The average particle size is estimated to be in the range of 13~25 nm. For comparison between pristine and modified NiFe_2_O_4_ surfaces, TEM of unmodified NiFe_2_O_4_ was characterized using TEM (see Figure 2d)

Additionally, the prepared materials were confirmed using the TEM diffraction pattern, as shown in Figure 2e. However, the miller indices were estimated for different d-spacing values in reference card JCPDS No.54-0964. EDAX estimated the elemental analysis of NFO@CNT. Thus, the EDAX shows the presence of Ni, Fe, O, and C. The elemental compositions of the NFO@CNT sample are shown in Figure 2f. Thus, the elemental percentage shown in the inset figure matches with our target structure of NiFe_2_O_4_, as the ratio between Ni and Fe is found as 1/2.

### 3.2. Electrochemical Characterization

**Figure 3:**
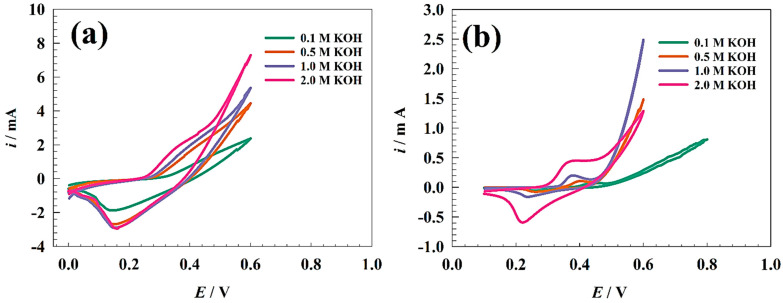
Comparison between (**a**) GC/NFO@CNT and (**b**) GC/NFO composites in different electrolyte concentrations.

The electrochemical studies of the modified GC/NFO and GC/NFO@CNT were studied in an alkaline medium. At the same time, the capacitive properties of the different modified electrodes were studied at different electrolyte concentrations. Since the capacitance performance of nickel-based electrodes in an alkaline medium mainly depends on the conversion of Ni(OH)_2_ to NiOOH, modified electrodes were activated in 1.0 M KOH using repeated CVs until the resultant current showed a stable response. Figure 3a,b show CVs of the modified GC/NFO and GC/NFO@CNT electrodes in a solution of KOH at different concentrations. One redox peak observed at (0.2 to 0.4 V (vs. Ag/AgCl)) is attributed to the Ni(OH)_2_/NiOOH redox couple. The increase in the oxidation/reduction current was observed due to the dependent of the ${OH}^{-}$ ions for the generation of redox species, according to the following equation [45,63]:

(2) $$6\mathrm{Ni}(\mathrm{OH}{)}_{2}+6\;OH^{-}↔6\mathrm{NiOOH}+6H_{2}O$$

Accordingly, the redox peak shifted to a more negative value by increasing the electrolyte concentration, and the electrochemical reaction tended to be more thermodynamically favored.

By comparison, the addition of CNT to the NFO nano-spinel oxide enhanced the faradic process by increasing available surface area and electrical conductivity. Furthermore, the metal-oxide-decorated CNT was reported to have efficient activity compared to pristine counterparts like ZnWO_4_@CNT, NiCo_2_O_4_@CNT, and Co_3_O_4_/CNT [64,65,66].

**Figure 4:**
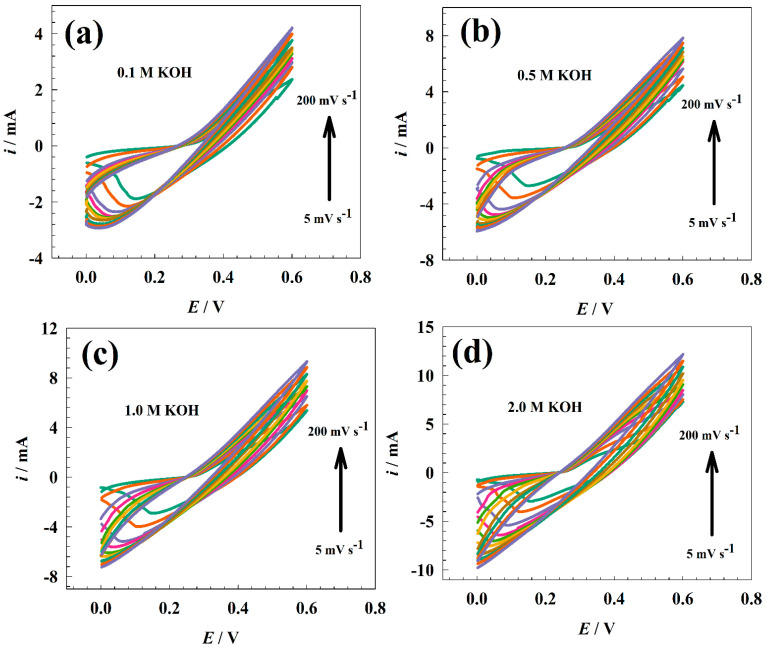
CVs of modified GC/NFO@CNT electrode at different concentrations of KOH.

Figure 4 shows the modified GC/NFO@CNT electrode CVs at different electrolyte concentrations (0.1 to 2.0 M KOH). The scan rate was changed in the range of 5–200 mV s^−1^. The diffusion coefficient of OH anions was studied using Randles–Sevcik equation:

*i*_p_ = 2.69 × 10^5^ × *n*^3/2^ × *A* × *D*^1/2^*C* × *ν*^1/2^ (3)

where *i_p_*: peak current, *A*: electrode geometrical area, *n*: electron participated in a redox reaction, *C*: concentration of KOH, *D*: diffusion coefficient, and *ν*: scan rate.

**Figure 5:**
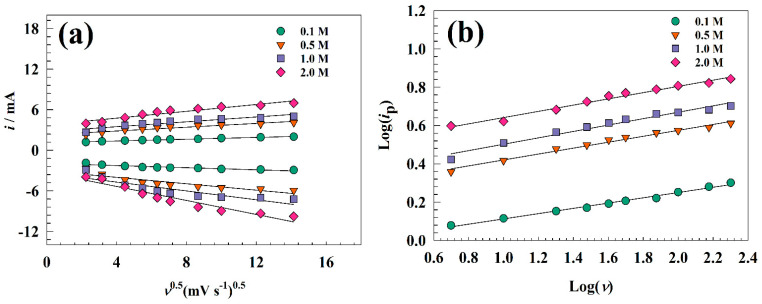
Representation of linear relations calculated for GC/NFO@CNT electrode. (**a**) Linear relation between the square root of scan rate and redox current. (**b**) Relation between the natural logarithm of both scan rate and oxidation current.

Accordingly, the diffusion coefficient for the modified electrode was estimated using the linear relation between the anodic and cathodic peak currents and the square root of the scan rate (see Figure 5a). The average diffusion coefficient was provided for each concentration as 1.65 × 1$0^{-5}$, 3.55 × 1$0^{-5}$, 4.62 × 1$0^{-5}$, and 6.41 × 1$0^{-5}$ cm^2^ s^−1^ for 0.1, 0.5, 1.0, and 2.0 M KOH, respectively. For the GC/NFO@CNT electrode, diffusion of OH ions increased versus concentration in the range of 0.1 to 1.0 M. In comparison, the diffusion at a concentration of 2.0 M is lower due to the columbic repulsion between the similarly charged ions.

For mixed capacitance mechanism, the following equation could be used [8,42]:

*i* = *s* × *ν*^*w*^ (4)

where *i* is current at characteristic potential, *ν* is sweep rate, and *s* and *w* are constants. However, the value of *w* represents the mechanism. The *w*-value indicates the charge storage mechanism with a value ranging from 0.5 to 1.0. Figure 5b shows the relation between the natural logarithm of scan rate and the natural logarithm of peak current. The values of *w* were provided as 0.54, 0.57, 0.67, and 0.68 for concentrations of 0.1, 0.5, 1.0, and 2.0 M, respectively. The estimated value of *w* indicates that the GC/NFO electrode storage mechanism tends to be mixed between bulk faradic and capacitive. Also, the increase in electrolyte concentration led to a shift in mechanism toward a more faradic process due to the availability of hydroxide ions in the solution, which is essential for the conversion of the redox system of Ni-based surfaces.

**Figure 6:**
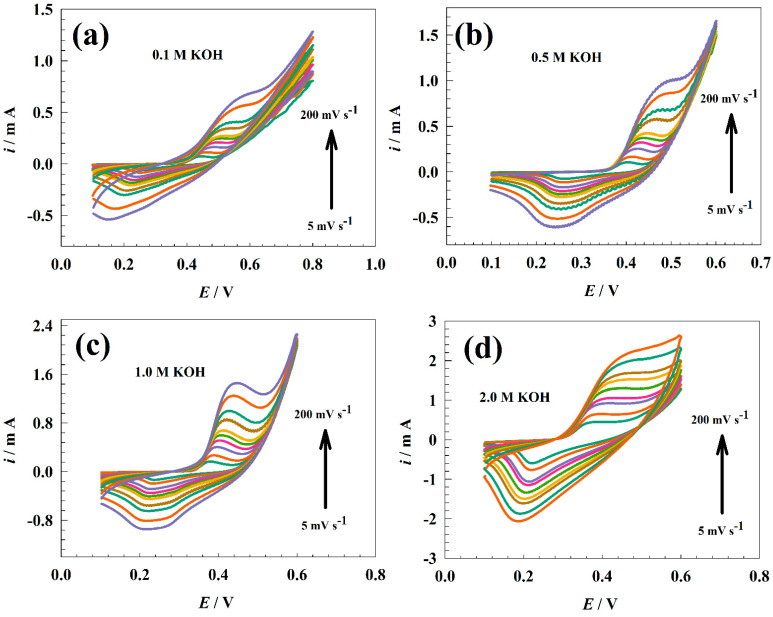
CVs of modified GC/NFO electrode at different concentrations of KOH.

The functionality of the modified GC/NFO electrode was examined in several KOH solutions. Figure 6 shows the CVs of the modified GC/NFO electrode at various scan rates (5 to 200 mV s^−1^) in various alkaline medium ranges (0.1 to 2.0 M). Increased scan rates of CNT-based composites impact the delineation of the NiOOH/Ni(OH)_2_ redox peak when compared to NFO@CNT. As opposed to the faradic current, the presence of CNTs increased the capacitive current.

**Figure 7:**
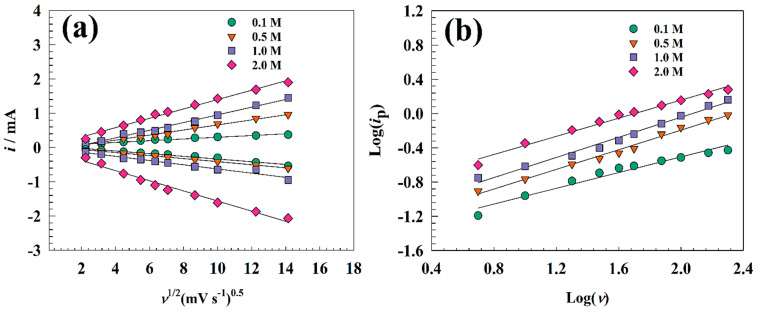
Representation of linear relations calculated for GC/NFO electrode. (**a**) Linear relation between the square root of scan rate and redox current. (**b**) Relation between the natural logarithm of both scan rate and oxidation current.

Since Randles–Sevcik relation should take the diffusion of ${OH}^{-}$ into account, the anticipated diffusion coefficients for modified GC/NFO electrodes are 6.33 × 1$0^{-6}$, 1.87 × 10^−5^, 2.84 × 1$0^{-5}$, and 3.44 × 1$0^{-5}$ cm^2^ s^−1^ for 0.1, 0.5, 1.0, and 2.0 M, respectively. For comparison, the mixed capacitance was calculated using the relation shown in Figure 7b and Equation (4). The slope of *w* was calculated to be 0.48, 0.51, 0.57, and 0.53 for electrolyte concentrations of 0.1, 0.5, 1.0, and 2.0 M, respectively. Therefore, the supplied value of the b slopes suggested that the bulk faradic process was used as the storage mechanism on the modified GC/NFO electrode.

**Figure 8:**
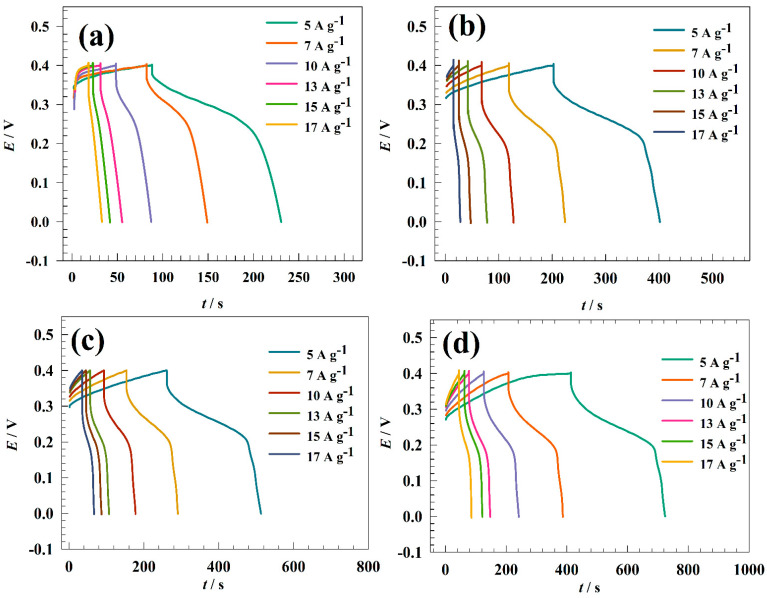
GCD of GC/NFO@CNT in a solution of (**a**) 0.1, (**b**) 0.5, (**c**) 1.0, and (**d**) 2.0 M KOH.

Galvanostatic charging and discharging techniques were used to characterize the charging and discharging of electrode surfaces. GCD techniques for modified GC/NFO@CNT at various electrolyte concentrations are shown in Figure 8a–d. To prevent oxygen evolution, the charging/discharging test occurred in the potential window of 0 to 0.4 V vs. Ag/AgCl. Otherwise, a charging current in the range of 5 to 17 A g^−1^ was picked. The charging and discharging times where the faradic process is accelerated by increasing the available ${OH}^{-}$ species can be increased by raising the KOH concentration. The modified electrode GC/NFO@CNT showed capacitance at 1.0 M KOH as 1169, 1007, 898, 800, 771, and 730 F g^−1^ for 5, 7, 10, 13, 15, and 17 A g^−1^, respectively. The computed capacitance for the modified electrode is listed in Table 1.

**Figure 9:**
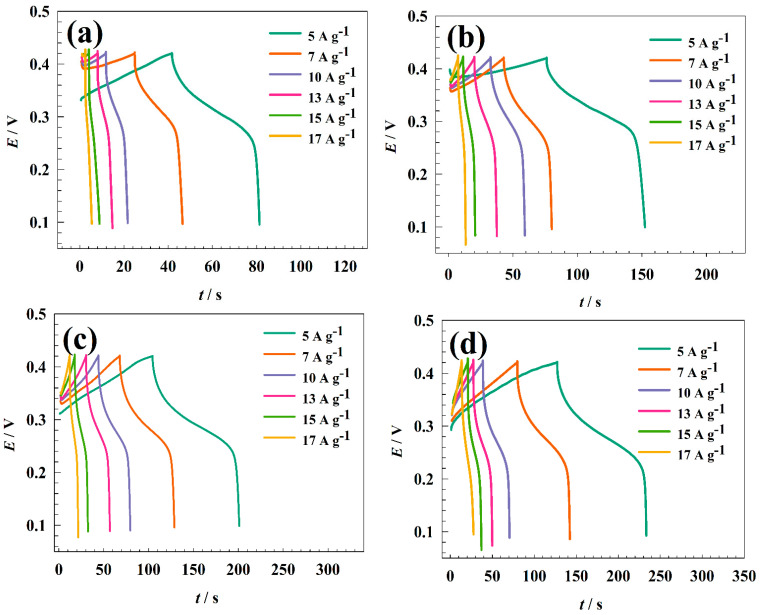
GCD of GC/NFO in a solution of (**a**) 0.1, (**b**) 0.5, (**c**) 1.0, and (**d**) 2.0 M KOH.

The charging–discharging ability was investigated in different KOH concentration ranges to compare CNT-modified and pristine NFOs. Figure 9 shows GCD curves of the GC/NFO electrode at different current ranges 5–17 A g^−1^ in ranges of KOH concentration (0.1 to 2.0 M). Specific capacitance was calculated for 1.0 M KOH concentrations as follows: 450, 366, 344, 328, 311, and 295 F g^−1^ for 5, 7, 10, 13, 15, and 17 A g^−1^, respectively. Thus, the increase in KOH concentration enhanced the capacitance of the modified electrode due to the enhancement of available OH ions that are essential for the NiOOH/Ni(OH)_2_ redox reaction.

**Figure 10:**
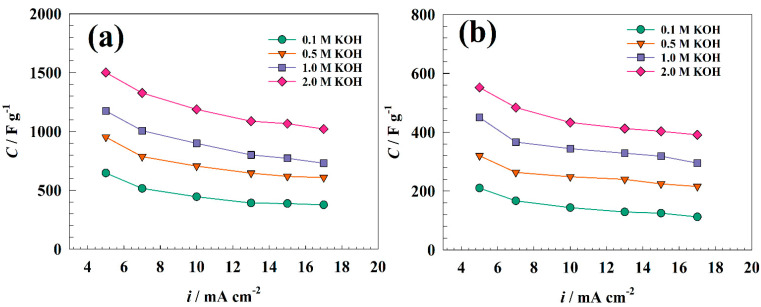
Relation between charging current and specific capacitance for (**a**) GC/NFO@CNT and (**b**) GC/NFO.

Figure 10 shows the relation between the charging current and capacitance of different electrode surfaces in various electrolyte concentrations. Thus, change in capacitance with the current was illustrated for GC/NFO@CNT (see Figure 10a). This is provided that rate capabilities for different electrolytes are 58, 63, 62, and 68% for 0.1, 0.5, 1.0, and 2.0 M, respectively. Figure 10b displays the relation between capacitance and current for modified GC/NFO electrodes. The rate capabilities are estimated as 53, 67, 65, and 70% for 0.1, 0.5, 1.0, and 2.0 M, respectively. Reduced charge and discharge times, which restrict charge diffusion in the films, may be owed to the drop in specific capacitance observed at high current densities [67].

**Figure 11:**
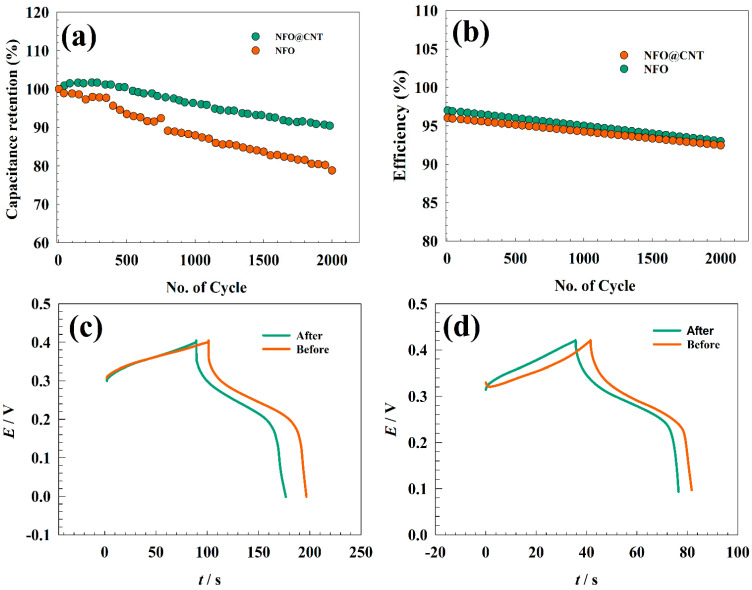
Study of different electrode durabilities for 2000 cycles (**a**) capacitance retention (%), and (**b**) columbic efficiency. Comparison between the 1st and the 2000th cycle of (**c**) GC/NFO@CNT and (**d**) GC/NFO electrodes.

Furthermore, the durability of the capacitor performance is considered an essential factor in judging the prepared materials. As represented in Figure 11a, the stability tests for modified GC/NFO@CNT and GC/NFO electrodes were carried out in a solution of 1.0 M KOH at the charging–discharging current of 10 A g^−1^ cm^−2^. For both electrodes, the capacitance retentions were estimated as 90.1 and 78.8% for GC/NFO@CNT and GC/NFO, respectively. Furthermore, the coulombic efficiencies of different modified electrodes were studied to evaluate the electrode performances. Figure 11b shows a slight decrease in efficiency after 2000 cycles. Thus, the GC/NFO@CNT efficiency decreased by 5% compared to 6.5% for GC/NFO counterparts.

The higher stability of CNT-modified composites regards outstanding properties of CNTs in capacitance applications. Higher adsorption/desorption of ${OH}^{-}$ on CNTs promotes the faradic process of Ni-based surfaces. Furthermore, GCD for the different modified surfaces is represented in Figure 11c,d. After repeated cycling, a lower IR drop was observed. Furthermore, there were higher charging and discharging times for activation of the additional Ni centers. The outcomes of the modified GC/NFO and GC/NFO@CNT were compared to other modified electrodes reported in the literature, as listed in Table 1.

**Figure 12:**
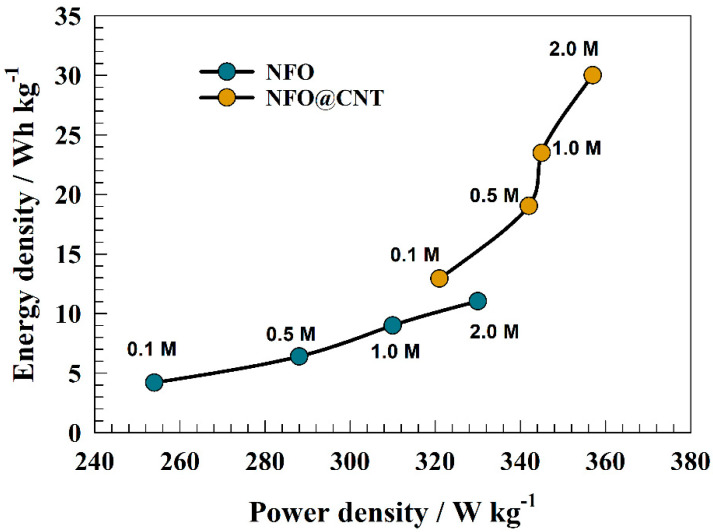
Ragone plot of GC/NFO and GC/NFO@CNT for different KOH conc. (0.1 to 2.0 M).

Additionally, modified GC/NFO@CNT and pristine GC/NFO were compared using a Ragone plot (Figure 12). The increases in both energy density and power density were observed with electrolyte concentration. Whereas, the highest power and energy densities were observed at 2.0 M of KOH. The provided values of energy density and power density were (11.2 Wh kg^−1^, 330 W kg^−1^) and (30.2 Wh kg^−1^, 357 W kg^−1^) for pristine and modified NFO, respectively.

**Table 1:** Capacitance performance comparison between GC/NFO and others reported in the literature.

Electrode	Preparation	Electrolytes	Potential Window (V)	*C_s_*/F g^−1^	Rate Capability	Stability (Cycle/Current)	Ref.
GC/NFO	Green method	1.0 M KOH	0.32	450 (5 A g^−1^)	65.1% (5 to 17 A g^−1^)	78.8% (2000 cycles at 10 A g^−1^)	This work
GC/NFO@CNT	Green method	1.0 M KOH	0.4	1169 (at 5 A g^−1^)	62.4% (5 to 17 A g^−1^)	90.1% (2000 cycles at 10 A g^−1^)	This work
Fe-MnO_2_	Hydrothermal	0.5 M Na_2_SO_4_	1.0	145 F g^−1^ (1 A g^−1^)	71.4% (1 to 10 A g^−1^)	95.9% (5000 cycles at 3 A g^−1^)	[68]
Ni–Co double hydroxide	Electrodeposition	6.0 M KOH	0.45	1246 F g^−1^ (1 A g^−1^)	91.8% (1 to 10 A g^−1^)	80.1% (1000 cycles at 10 A g^−1^)	[69]
CoNiFe-layered double hydroxide	In situ growth method	6.0 M KOH	0.4	1203 F g^−1^ (1 A g^−1^)	77.1% (1 to 10 A g^−1^)	94% (1000 cycles at 20 A g^−1^)	[70]
MnO_2_@CNTs/CNTs	Vacuum filtration	1 M Na_2_SO_4_	1.0	149 (0.2 A g^−1^)	85% (0.2 to 5 A g^−1^)	90% (5000 cycles at 50 mV s^−1^	[71]
Fe-MnO_2_@ CNF	Chemical	1 M Na_2_SO_4_	1.0	210 (0.3 A g^−1^)	83% (0.3 to 10 A g^−1^)	94% (4500 cycles at 2 A g^−1^)	[72]
RGO/Fe_2_O_3_	Chemical	2.0 M KOH	1.1	469.5 (4 A g^−1^)	49% (4 to 8 A g^−1^)	88% (5000 cycles at 8 A g^−1^)	[73]

**Figure 13:**
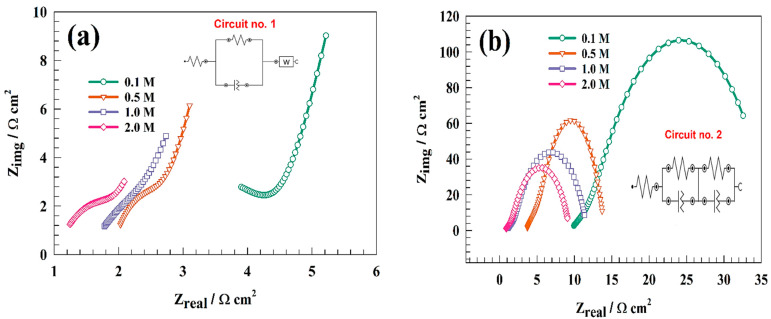
Nyquist plots of (**a**) GC/NFO@CNT and (**b**) GC/NFO electrodes at different electrolyte concentrations.

The improvement of the modified surface was obtained using the electrochemical impedance technique. Comparative studies were employed to find out the effect of the addition of CNTs to an NFO composite. Thus, Figure 13a represents a Nyquist plot of the modified GC/NFO@CNT electrode for different electrolyte concentrations. The shift in starting impedance values is attributed to a decrease in solution resistance with electrolyte concentrations. Additionally, the obtained EIS data for the GC/NFO@CNT surface indicated the mixing of charge transfer and diffusion processes. Whereas, the EIS data fitting uses NOVA software, as referenced in Figure 13a inset. The equivalent circuit deduced for GC/NFO@CNT contained two resistances referring to solution resistance and charge transfer resistance. The presence of a constant phase element indicated the surface roughness or inhomogeneous distribution over the electrode surface. Relatedly, diffusion elements are connected in series with charge transfer cell. The fitting parameters for the modified GC/NFO@CNT surface are listed in Table 2.

Figure 13b shows Nyquist plots of EIS measurements of GC/NFO in different concentrations of KOH. EIS results were fitted using NOVA software. The diameter of the semi-circuit Nyquist plot reflects the electrode’s activity toward the faradic redox process. Thus, increasing the concentration of the supporting electrolytes shifted the solution resistance toward a lower value. Therefore, provided solution resistances for the GC/NFO electrode were 4.14 and 2.28 Ω cm^2^ for 0.1 and 2.0 M KOH, respectively.

Additionally, Nyquist data were fitted as represented in the circuit-illustrated inset in Figure 13b. The fitting circuit included Rs, corresponding to the resistance of the solution, R_1_, and R_2_, attributed to the outer and inner layer resistances, respectively. Relatedly, Q_1_ and Q_2_ can be established as constant phase elements for the outer and inner surfaces, respectively. The estimated EIS parameters for modified GC/NFO electrodes in different KOH concentrations are listed in Table 3. Whereas, higher KOH concentrations lead to higher faradic currents and consequently lower charge transfer resistances.

## 4. Conclusions

The capacitance of nickel–iron-based spinel oxide in an alkaline medium was estimated. Comparative studies between pristine NiFe_2_O_4_ and NiFe_2_O_4_@CNT showed the dramatic effect of CNTs as carbon support for enhancing the faradic process of nickel. The extended surface area, along with high electron transfer, facilitated the Ni(OH)_2_/NiOOH redox process. The diffusion coefficients utilized by Randles–Sevcik equation were 2.84 × 10^−5^ and 4.62 × 10^−5^ cm^2^ s^−1^ for pristine and modified NiFe_2_O_4_ electrodes.

The effect of electrolyte concentrations was studied for pristine and CNT-modified NiFe_2_O_4_. Whereas, the capacitance increased by 46 up to 82% by increasing the electrolyte from 0.1 to 2.0 M KOH.

On the other hand, the capacitance rate capability was observed as 65.1% (5 to 17 A g^−1^) and 62.4% (5 to 17 A g^−1^) for NiFe_2_O_4_ and NiFe_2_O_4_@CNT electrodes. Both pristine and CNT-modified surfaces showed high stability after 2000 cycles. Furthermore, the redox process was estimated using EIS. The charge transfer resistances were estimated in 2 M KOH as 3.43 and 12.79 Ω cm^2^ for GC/NiFe_2_O_4_@CNT and GC/NiFe_2_O_4_, respectively.

## Figures and Tables

**Table 2:** EIS parameters of modified GC/NFO@CNT electrode at different electrolyte concentrations.

KOH (M)	*R_s_*	*R*_1_	*Q_1_*		*W*
	*R* ( Ω cm^2^)	*R* (Ω cm^2^)	Y0 (Ω^−1^cm^2^s^−^*^n^*)	*n*	Y0 (Ω^−1^cm^2^s^−^*^n^*)
0.1	6.43	10.14	0.000656	0.1181	0.11318
0.5	3.71	6.14	0.012638	0.37549	0.16978
1.0	4.14	5.42	0.029051	0.23167	0.29914
2.0	2.28	3.43	0.019652	0.27925	0.54293

**Table 3:** EIS parameters of modified GC/NFO electrode at different electrolyte concentrations.

KOH (M)	*R_s_*	*R*_1_	*R*_2_	*Q*_1_		*Q*_2_	
	*R* (Ω cm^2^)	*R* (Ω cm^2^)	*R* (Ω cm^2^)	Y0 (Ω^−1^cm^2^s^−^*^n^*)	*n*	Y0 (Ω^−1^cm^2^s^−^*^n^*)	m
0.1	12.11	17.11	40.15	0.0013199	0.2482	0.0043451	0.75486
0.5	6.87	8.87	19.21	0.0025709	0.31794	0.00089369	0.76123
1.0	1.96	2.95	14.96	0.0026587	0.40007	0.00079411	0.73669
2.0	1.46	2.65	12.79	0.002747	0.63216	0.0009814	0.81583

## Data Availability

The datasets used and analyzed during the current study are available from the corresponding author upon reasonable request.
